# A Bioglass-Based Antibiotic (Vancomycin) Releasing Bone Void Filling Putty to Treat Osteomyelitis and Aid Bone Healing

**DOI:** 10.3390/ijms22147736

**Published:** 2021-07-20

**Authors:** Raquib Hasan, Kambri Schaner, Pranothi Mulinti, Amanda Brooks

**Affiliations:** 1 Department of Pharmaceutical Sciences, North Dakota State University, Fargo, ND 58102, USA; pranothimulinti@gmail.com; 2 Department of Biological Sciences, North Dakota State University, Fargo, ND 58102, USA; kambri.schaner@ndsu.edu; 3 Department of Research and Scholarly Activity, Rocky Vista University, Ivins, UT 84734, USA

**Keywords:** osteomyelitis, vancomycin, bone void filler, bioglass, 45S5 bioactive glass, controlled drug delivery, bone regeneration, orthopedic surgery, composite bone biomaterials, *Staphylococcus aureus*

## Abstract

While the infection rate after primary total joint replacements (TJR) sits at 1–2%, for trauma-related surgery, it can be as high as 3.6 to 21.2% based on the type of trauma; the risk of reinfection after revision surgery is even higher. Current treatments with antibiotic-releasing PMMA-based bone cement/ beads and/or systemic antibiotic after surgical debridement do not provide effective treatment due to fluctuating antibiotic levels at the site of infection, leading to insufficient local antibiotic concentration. In addition, non-biodegradable PMMA does not support bone regrowth in the debrided void spaces and often must be removed in an additional surgery. Here, we report a bioactive glass or bioglass (BG) substrate-based biodegradable, easy to fabricate “press fitting” antibiotic-releasing bone void filling (ABVF-BG) putty to provide effective local antibiotic release at the site of infection along with support for bone regeneration. The ABVF-BG putty formulation had homogenously distributed BG particles, a porous structure, and showed putty-like ease of handling. Furthermore, the ABVF-BG putty demonstrated in vitro antibacterial activity for up to 6 weeks. Finally, the ABVF-BG putty was biodegradable in vivo and showed 100% bacterial eradication (as shown by bacterial cell counts) in the treatment group, which received ABVF-BG putty, compared to the infection control group, where all the rats had a high bacterial load (4.63 × 10^6^ ± 7.9 × 10^5^ CFU/gram bone) and sustained osteomyelitis. The ABVF-BG putty also supported bone growth in the void space as indicated by a combination of histology, µCT, and X-ray imaging. The potential for simultaneous infection treatment and bone healing using the developed BG-based ABVF-BG putty is promising as an alternative treatment option for osteomyelitis.

## 1. Introduction

Despite advances in both surgical techniques and pharmaceutical treatments, acute and chronic osteomyelitis, commonly caused by *Staphylococcus aureus* [1,2,3], remains one of the most difficult orthopedic conditions to treat. There has been a steady increase of bone and joint infections due to high energy trauma and as a consequence of surgical procedures such as bone fracture surgery, total joint replacements, etc., which may lead to chronic osteomyelitis [2,3]. Unfortunately, due to an inadequate penetration of antibiotics in poorly vascularized and necrotic bone, repeated surgical interventions are often necessary [4]. Surgical interventions may include the removal of all implants as well as aggressive debridement of infected or necrotic bone and any severely affected soft tissues [5]. Following the initial implant removal and debridement, it is equally important to fill up remaining void spaces in the bone, particularly that abutting replacement hardware, to prevent further infection. Consequently, antibiotic-loaded polymethylmethacrylate (PMMA)-based bone cement (ALBC), along with systemic antibiotic therapy for 4–6 weeks, has become the clinical standard of care to treat these infections. Unfortunately, despite the initial enthusiasm for the technology, ALBC suffers from several drawbacks, including the potential degradation of antibiotics due to high polymerization temperature, the necessity for a subsequent removal surgery due to non-biodegradability, and the possibility of biofilm formation on the ALBC surface due to subtherapeutic levels of antibiotic release after burst release is exhausted (generally 2 weeks or less) [6]. Alternatively, a variety of bone graft materials, some of which are capable of supporting bone regeneration, currently satisfy this space-filling role; unfortunately, despite their promises, many of them suffer from substantial rates of failure due to both the porosity that provides a haven for bacterial growth and faster resorption in the presence of persistent local infection [4]. Due to the issues with ALBC as well as with natural bone grafts as outlined above, bone graft substitutes are gaining acceptance as a promising replacement. Bone graft substitutes have several advantages over natural bone graft materials, including no need for harvesting, unlimited availability, support for bone healing, biodegradability, and ability to act as a predictable and consistent pharmaceutical carrier [2,7].

Conversely, BG, which has slowly gained acceptance since its introduction over 50 years ago for its ability to seamlessly bond to bone and facilitate healing, has not been used as antibiotic carrier [8]. BG has been shown to convert to hydroxyapatite (HA) in vivo as well as to have osteoconductivity and strong bond formation with bone [9,10,11]. Several types of bioglass have been previously investigated such as silicate glasses (using SiO_2_ as the glass network former), borate glasses (using B_2_O_3_ as the glass network former) and phosphate glasses (using P_2_O_5_ as the glass network former) [12]. Out of these, silicate-based bioglass 45S5 has been studied extensively for its bioactive and bone-healing properties. The composition of 45S5 bioglass is 45% SiO_2_–24.5% Na_2_O–24.5% CaO–6% P_2_O_5_ (%wt/wt). 45S5 bioglass is optimized for bioactivity and is known for its enhanced bioactivity, its influence on stem cell differentiation into osteoblasts, and favoring well-mineralized bone matrix formation [13]. In the current study, 45S5 bioglass was chosen for its bone-healing properties as the bone graft substitute material in the antibiotic-releasing, bone void filling putty (ABVF-BG).

Prior studies using bioactive glass particles to treat osteomyelitis were supplemented with systemic antibiotic therapy [14,15]. Combining the success of BG with the promise of using bone graft substitute as a drug carrier leads to a supposition that a BG–polymer composite may prove to be a particularly effective antibiotic delivery system where the polymer component would carry antibiotic and control the drug release while the BG substrate would support bone tissue regeneration. Thus, the focus of the current study is to develop a biodegradable BG-based, antibiotic (vancomycin)-releasing, bone void filling putty (ABVF-BG) that provides antibiotic release for 4 to 6 weeks to treat osteomyelitis, is easily press-fitted into a bone void space and provides support for bone growth. In the current study, fabrication of ABVF-BG putty and physical characterization of the putty was carried out. In vitro and in vivo antibacterial activity and bone-healing property were also assessed.

## 2. Results

### 2.1. X-ray Diffraction (XRD) of Bioglass (BG)

**Figure 1:**
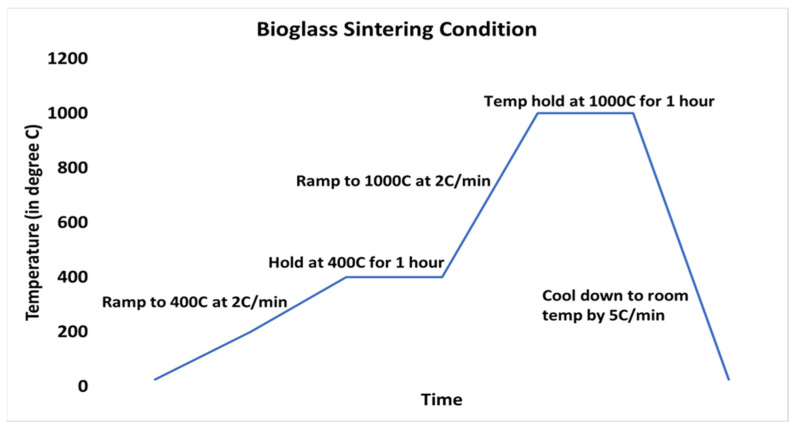
Profile of the heating and sintering temperatures during BG fabrication.

**Figure 2:**
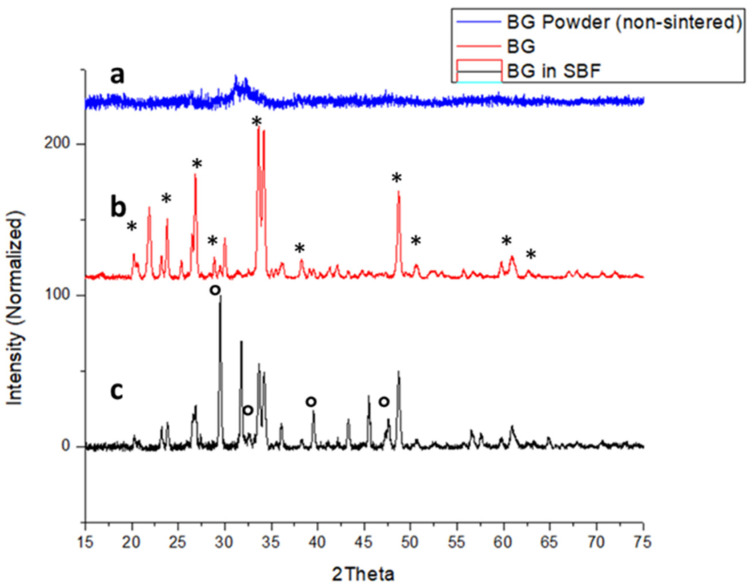
(**a**) XRD of amorphous BG particles before sintering. (**b**) XRD of partially crystalline BG after sintering. Major peaks corresponding to the crystalline phase containing Na_2_Ca_2_Si_3_O_9_ are marked with *. (**c**) Sintered bioglass after been immersed in simulated body fluid for 4 days. Presence of HA can be seen, as indicated by °.

Bioglass was sintered by using amorphous 455S bioglass powders at conditions outlined in Figure 1; details can be found in the method in Section 4.2. XRD was done to assess the crystallinity of sintered BG. XRD of non-sintered BG shows amorphous XRD spectra (Figure 2a). The sintered BG showed a partially crystalline structure with crystalline peaks related mostly to crystalline Na_2_Ca_2_Si_3_O_9_ (Figure 2b) [16,17]. The BG particles exposed to simulated body fluid (SBF) showed the presence of hydroxyapatite (HA) formation (Figure 2c). It should be noted from the changing XRD pattern of the SBF exposed BG particles that there was reduced crystallinity compared to sintered-only BG particles (Figure 2b).

### 2.2. Scanning Electron Microscopy (SEM) and Energy-Dispersive X-ray Spectrometry (EDS) of Sintered BG and ABVF-BG Putty

**Figure 3:**
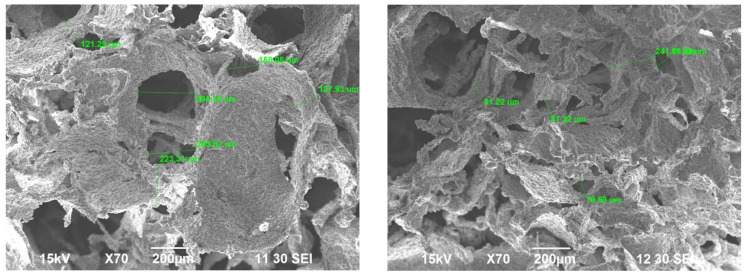
SEM image of BG scaffold. The porous structure is visible in the images. The green labels indicate pore measurements.

**Figure 4:**
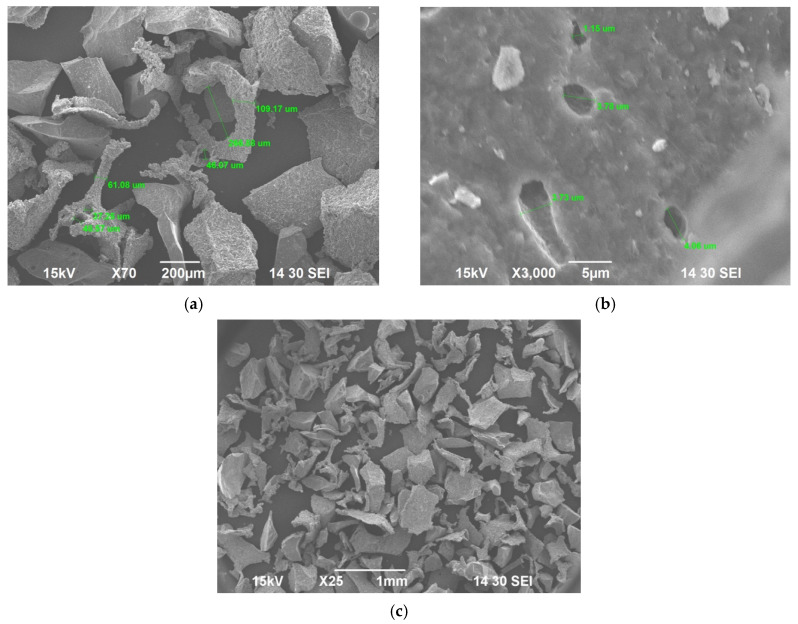
(**a**) SEM image of crushed BG. Struts of different width are seen; (**b**) The densified strut morphology and micropores are also visible on the struts. (**c**) Crushed BG particles. The green labels indicate strut and pore measurements.

**Figure 5:**
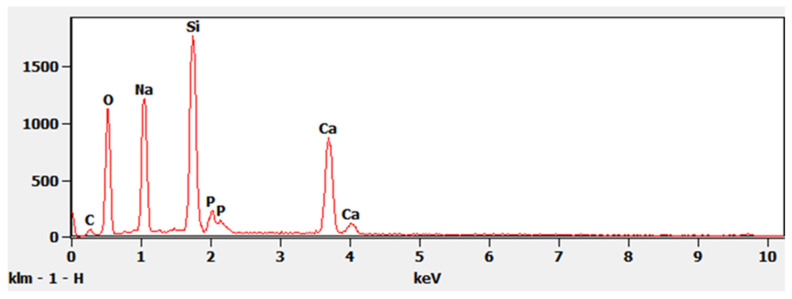
EDS spectra showed the presence of Na, Si, P, and Ca in the sintered BG.

**Figure 6:**
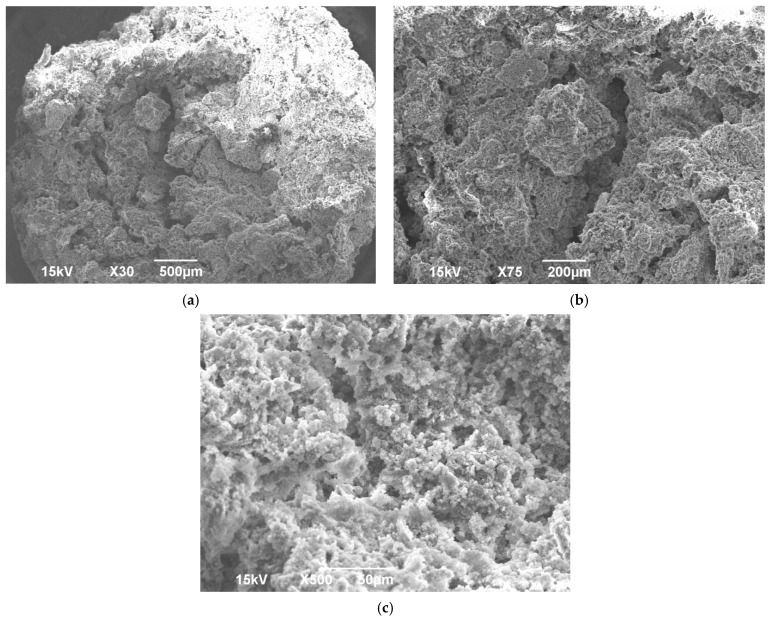
SEM showing the (**a**) outer structure of the ABVF-BG putty and (**b**,**c**) inner porous structure.

SEM was done to evaluate both the porosity of BG scaffold, the morphology of the scaffold and the morphology of the crushed BG particles. EDS was also run on crushed BG particles (150 to 425 μm). SEM of sintered BG scaffold showed a porous structure with pores ranging from 80 to 223 μm (Figure 3). The strut of the scaffold structure ranged from 81 to 242 μm (Figure 3). The strut, which resulted from the partial crystallinity of the sintered BG, created a scaffold-like structure and may increase the mechanical strength of the structure [16]. SEM of morselized, sintered BG showed particles of different sizes and width (Figure 4a,c), corresponding to the struts of different width seen in the sintered BG scaffold (Figure 3). A close image of the strut showed densification after sintering along with micropores on the particles ranging from 1 to 4 μm (Figure 4b). EDS spectra of the sintered BG showed the presence of Ca, P, Na, and Si in the sintered BG (Figure 5), which corresponds to the elements present in the BG powder constitutes used to prepare the sintered BG scaffold. Importantly, SEM of the ABVF-BG putty with sintered BG particles showed a porous inner structure (Figure 6).

### 2.3. Micro-Computed Tomograpgy (μ-CT) of ABVF-BG Putty

**Figure 7:**
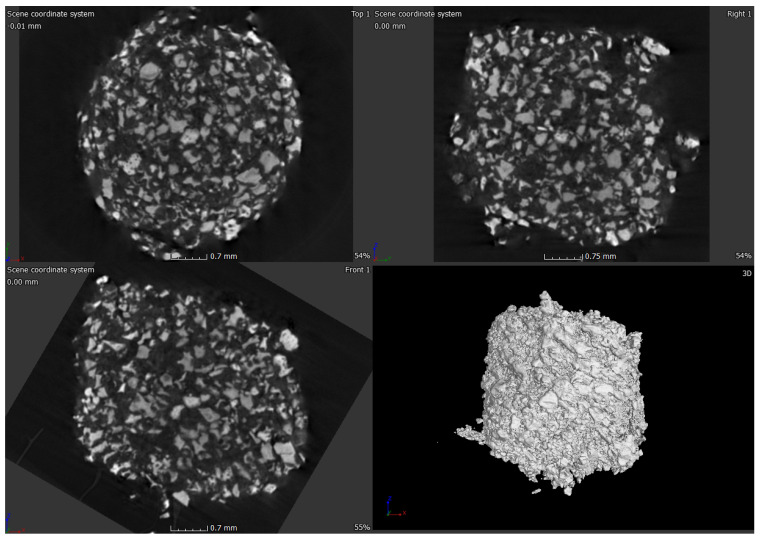
μ-CT image of ABVF putty. BG particles appear to be homogenously distributed in the polymer matrix.

μ-CT was done on the ABVF-BG putty to assess the distribution of BG particles in the polymer matrix of ABVF-BG. μ-CT of the ABVF-BG putty showed a homogenous distribution of BG particles embedded in the polymer matrix (Figure 7). The BG particles occupy 53.46% (*v/v*) volume in the ABVF-BG polymer matrix.

### 2.4. In Vitro Antimicrobial Assay

**Figure 8:**
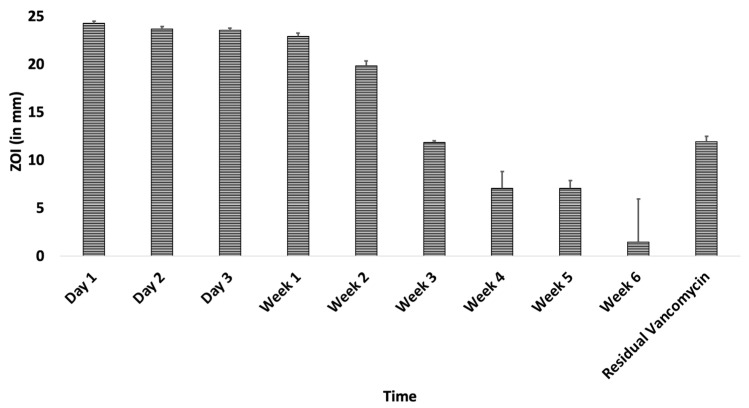
Zone of inhibition of released vancomycin from ABVF-BG putty against *S. aureus* (strain ATCC 49230). Released antibiotic at different timepoints showed bacterial killing. Residual antibiotic also showed bacterial killing.

Each ABVF-BG putty weighed 93.5 mg ± 2 mg and contained a total of 22.2 mg of vancomycin on average as prepared. Vancomycin released from ABVF-BG putty showed efficient antibacterial activity against *S. aureus* for up to 6 weeks (Figure 8), although the standard deviation of the ZOI was large at week 6. Furthermore, the residual vancomycin extracted from ABVF-BG after 6 weeks showed a ZOI, indicating that there was still some residual vancomycin left in the putty after 6 weeks, indicating that the release could extend beyond 6 weeks.

### 2.5. In Vitro Cell Viability Assay

**Figure 9:**
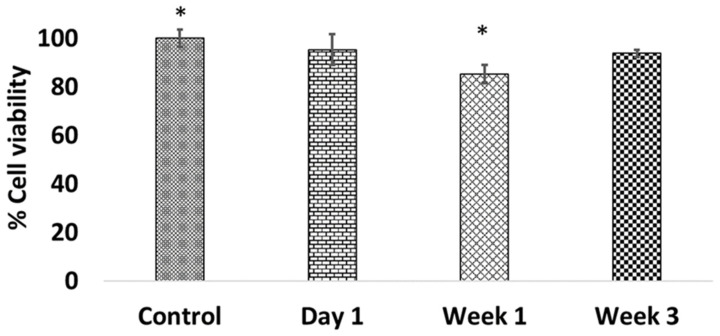
No significant difference was seen in cell viability when cells were exposed to day 1 and week 3 drug-released media, although a significant drop in cell viability was observed when cells were exposed to week 1 drug release sample. *n* = 3 for each group, * *p* < 0.05.

MG-63 osteoblast cells were exposed to drug-released media from ABVF-BG putty to assess the cytotoxicity of released vancomycin at different time points. Compared to control cells, which were not exposed to any vancomycin, no significant change in cell viability was observed when cells were exposed to day 1 and week 3 drug-released media (Figure 9). However, cell viability dipped to just over 80% when exposed to week 1 drug-released media, which is a significant decrease (*p* < 0.05) in viability compared to the control (Figure 9).

### 2.6. In Vivo Rat Infection Model

A rat osteomyelitis model was used to assess the in vivo performance of ABVF-BG according to approved protocol by the Institutional Animal Care and Use committee at North Dakota State University (protocol—A19019).

#### 2.6.1. X-ray and μ-CT of Rat Bones

**Figure 10:**
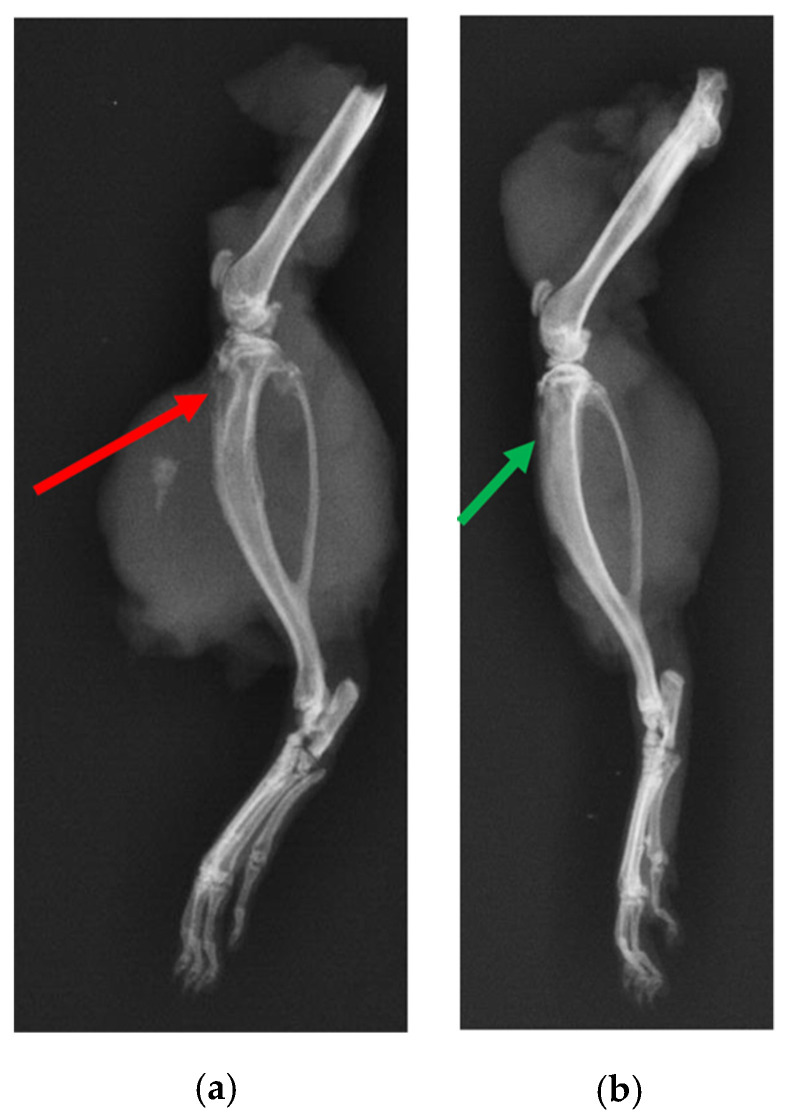
X-ray of rat tibia. (**a**) The red arrow indicates osteomyelitic bone in the control rat. (**b**) Alternatively, the green arrow shows healed bone in the treatment group.

**Figure 11:**
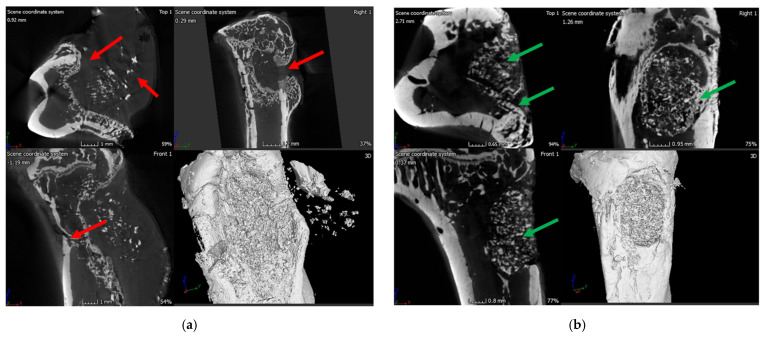
(**a**) μ-CT images of the rat bone in the infection control group showed signs of infection: narrowing of marrow space, presence of puss-filled fibrous capsule, sinus tract, and deformed bone with ectopic bone growth (red arrows); (**b**) The treatment group rat bone μ-CT images is showing signs of healing bone as well as the formation of cortical and cancellous bone in the space where the hole was drilled (green arrows).

X-ray of the tibia in the infection control group (i.e., BG containing putty with no antibiotic) showed classic signs of infection such as un-healed, osteolytic, and deformed bone (Figure 10). A narrowing of marrow space was also visible. In contrast, the tibia of the treatment group (ABVF-BG putty with vancomycin) showed no signs of infection, and the bone appeared to have healed (Figure 10). μ-CT images showed the presence of infection in the control rat tibia (Figure 11a), including signs of osteomyelitis such as narrowing of the marrow space, the presence of a puss-filled fibrous capsule, sinus tracts, and deformed bone with ectopic bone growth. Alternatively, healing bone without signs of infection was seen in the μ-CT images of tibia from the treatment rats. Specifically, the drilled hole at the surgical site was being filled by immature cancellous and cortical bone (Figure 11b).

#### 2.6.2. Bacterial Colony Count

**Figure 12:**
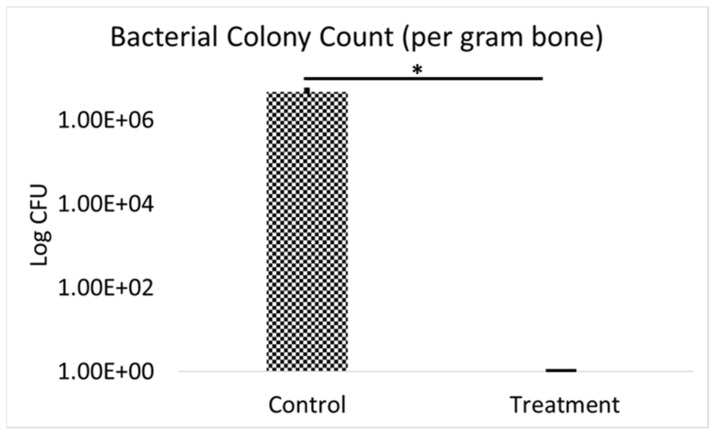
A high amount of bacterial presence was seen in infection control group rat tibiae. The treatment group rat bones showed no bacterial colony in culture. * *p* < 0.002.

The bacterial colony count confirmed the infection visible in imaging (Figure 10a and Figure 11a) of the control group rats. The control group rat bones showed a high bacterial load (4.63 × 10^6^ ± 7.9 × 10^5^ CFU/gram bone) (Figure 12) when compared to the tibiae from the treatment group, which showed no bacterial growth in culture confirming the absence of any infection (Figure 12).

#### 2.6.3. Histology

**Figure 13:**
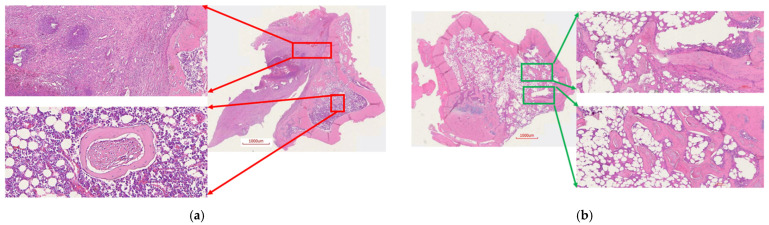
H&E staining of rat bone. (**a**) Control rat bone. Note that there was growth with narrowing of the marrow space. Additionally, there were places of bone thickening and a large fibrous capsule with inflammatory cells; (**b**) Bone from the treatment group rat showed ongoing healing of the bone, including new bone formation in the defect. Osseointegratation of the host bone was seen, as the degradation of the ABVF-BG allowed new bone in-growth.

To assess the histological features of the bones at the surgical site, H&E staining was done. Tibia from the control rats showed unhealed bone, including a lack of new bone growth and the presence of fibrous tissue, which is a consequence of infection. Additionally, the presence of chronic infection was evident (red arrows, Figure 13a). On the contrary, bone from the treatment group showed ongoing bone healing, including new bone growth and osseointegration, likely resulting from the absence of infection (Figure 13b). It also showed limited mature collagen structure (green arrows, Figure 13b). These signs are consistent with the documented healing process of bone in the absence of infection.

## 3. Discussion

In recent years, bioactive glass (BG) has garnered much attention for its biological activity, especially in the case of bone tissue engineering. Since its first inception, a variety of BG has been prepared and tested for its impact on bone and soft tissue engineering, of which 45S5 is the most widely studied [18,19]. In today’s market, most of the commercially available BG is used for trauma and treatment of bone injuries [20]. 45S5 BG is regarded as the gold standard in bone regenerative repair due to its bioactivity, biodegradability, and bone-bonding properties [17]. BG shows not only higher quantity and quality of bone formation compared to synthetic hydroxyapatite (HA) [21] but also aids a faster onset of bone formation [22]. These properties may be a result of a higher degradation rate when compared to HA [23], which is desirable to make space for growing bone [20]. In our study, we have seen the BG particles start to degrade, as evidenced by the decrease in crystallinity after 4 days of immersion in SBF (Figure 2c), as well as by an indication of HA formation (Figure 2c). Moreover, ABVF-BG putty showed new bone formation and bone healing in an in vivo study (Figure 10b, Figure 11b and Figure 13b). The rate of new bone formation in the drilled area was seemingly higher when ABVF-BG was used in bone defects compared to our prior study where ABVF contained hydroxyapatite and calcium carbonate-based particles as a bone graft substitute in other projects in our lab, according to preliminary findings (Supplementary Figure S2).

Bioactive glass or BG has been a subject of interest in tissue engineering, specifically for orthopedic applications, because of its bone-bonding properties [24]. The 45S5 amorphous BG powder, used in this study to synthesize sintered BG scaffold, is particularly well known for its ability to bond bone. Its ability to bond bone can be attributed to the conversion of the BG material to a hydroxyapatite (HA)-like layer. The conversion starts with the formation of a SiO_2_-rich gel on the 45S5 glass surface by ion exchange reactions. Dissolution and diffusion of the ions from the BG through the SiO_2_-rich gel layer, contribute to the reaction between Ca^2+^ and PO_4_^3–^ ions from the surrounding liquid. This reaction ends in the growth of an HA-like layer on the gel surface [25].

BG is also known to upregulate different genes such as insulin-like growth factor II (IGF-II), which induces osteoblast proliferation along with an increase in secretion of the extracellular matrix (ECM) [26,27]. The upregulation may be due to the ions such as calcium (Ca) and silicon (Si) released from BG [28]. Moreover, ions such as Si, Ca, and inorganic phosphate (Pi) play an essential role in bone tissue regeneration. Si plays a critical role in the formation and calcification of bone tissue by HA precipitation, collagen I formation, and osteoblast differentiation [29,30,31]. Ca ions increase ECM formation, osteoblast differentiation and proliferation, and IGF activity [32,33]. Pi ions increase matrix Gla protein (MGP) expression, which regulates bone formation [34]. In the current study, the BG particles showed the presence of Ca, Pi, and Si ions, as was seen in the EDS spectra (Figure 5). The presence of these ions in BG particles may have led to new bone formation when ABVF-BG was implanted in the bone defect (Figure 11b and Figure 13b)

The ABVF-BG putty, when implanted in infected bone defect, showed new bone formation and osseointegration after 8 weeks from implantation (Figure 10b, Figure 11b, and Figure 13b). Perhaps as a consequence of its ability to bond bone, BG-based bone void fillers, particularly when formulated as a putty, have previously been shown double the amount of bone formation after 6 weeks when compared to particulate BG in a sheep vertebral defect model [35]. Importantly, the putty matrix may have allowed more space for bone formation as the polymer matrix degraded than what tightly compacted particulate would allow. Furthermore, a putty might prove preferable for both its handling characteristics as well as its immune interactions, as the migration of loose BG particulates has been shown to provoke inflammation [36]. Although seen in the control group, the treatment group animals did not show such presence of inflammatory cells in H&E staining (Figure 13).

To take advantage of the bone bonding and healing properties of BG in the current study, amorphous 45S5 BG powder was sintered to create a partially crystalline scaffold. Using the conditions described here (Section 2.3) yielded the presence of Na_2_Ca_2_Si_3_O_9_ in the crystalline region after sintering (Figure 2b) when compared to the amorphous 45S5 BG powder (Figure 2a). The presence of Na_2_Ca_2_Si_3_O_9_, which was shown to be converted into an amorphous form in the presence of phosphate ions, is critical for the bioactivity of any BG-based scaffold [16]. Previously, the bioactivity of Na_2_Ca_2_Si_3_O_9_ crystals has been reported, showing that the combination of this non-phosphate bioactive crystal with phosphate ions from the BG particulate leads to faster HA formation in vitro in SBF, similar to amorphous BG powder [37]. In a previous study, the rate of HA crystal formation on crystalline BG particulate was slower when put into SBF, but the bioactivity was still preserved [38]. In the current study, after subjecting the sintered BG particles to SBF, peaks corresponding to HA appeared in the XRD spectrum. A reduced crystallinity of Na_2_Ca_2_Si_3_O_9_ was also observed (Figure 2c), which may indicate that the crystalline Na_2_Ca_2_Si_3_O_9_ phase had started to convert to a more amorphous form [16]. Nonetheless, bioactivity was preserved, as was demonstrated by the new bone formation seen in the in vivo study described in the current research.

In addition to the traditional, albeit slightly slower, changes in the surface of the BG that leads to bone bonding and likely contribute to bone healing, the influence of porosity in this process cannot be neglected. In our current study, the sintered BG scaffold had a porous structure with varying pore sizes (Figure 3); this porosity was likely a result of the sacrificial polyurethane polymer template, although the variability of the pore sizes may reflect the partial structural collapse of the BG scaffold in the absence of the polymer template during the sintering process. Alternatively, variability in pore size may reflect incomplete penetration of BG slurry in the polyurethane template. Nevertheless, the range of pore sizes seen may prove important for vascularization and bone ingrowth. Different studies reported different pore sizes as optimal, but there is no clear consensus. Pore size ranges of 150–710 μm for bone tissue engineering [39], 5–600 μm for bone regeneration [40], 50 to 710 μm for bone regeneration, and 5 μm for neovascularization [41] have been reported. Although there is much variation in reports, it can be said that porous structure is necessary for bone tissue healing [42]. Although we did not use the sintered BG scaffold, nevertheless, the porous structure of the scaffold may be explored and utilized for other bone-tissue engineering purposes. In our study, we used crushed particles made from the sintered BG scaffolds (Figure 4a). Nonetheless, the ABVF-BG containing the particles showed a porous structure (Figure 6), which might have played a role in new bone formation in the in vivo study (Figure 11b). An SEM image of the crushed BG scaffold showed the particles used to formulate the ABVF-BG (Figure 4a). A zoomed-in view showed a densified structure of the particles with micropores, as noted in earlier studies [16]. Densification here is desired, as this would provide a measure of structural integrity under compression. The EDS of the sintered BG confirmed the presence of the elemental composition in the sintered BG (Figure 5) as was present in the amorphous 45S5 BG powder used. Additionally, ABVF-BG putty prepared with sintered BG particles showed a porous macrostructure throughout the composition (Figure 6b) as well as the presence of micropores throughout the structure (Figure 6c). Macroporosity likely resulted from the disbursement of sintered BG particles in a putty-like polymer matrix. The μ-CT of the ABVF-BG putty showed the homogenous distribution of the BG particles embedded in the polymer matrix (Figure 7). The packing density of the particles in the putty is a critical parameter that can be optimized to facilitate host bone cell infiltration and ingrowth. The BG-based ABVF-BG putty reported here has an appropriate spatial distribution of the BG particles in a degrading polymer matrix, which may be further optimized, offering a distinct advantage of our ABVF-BG putty over other BG particulate-only fillers [35]. Ultimately, the presence of both a macro and micro-pores as seen in the produced ABVF-BG putty would support bone tissue healing. In addition to that, the particle size distribution of the BG used in the AVBF-BG putty ranged from 175 to 425 μm; according to previous reports, bone graft substitute particle size ranges from 100 to 500 μm helped in more new bone formation compared to when smaller (<105 μm) or larger (1000–2000 μm) particle sizes were used [43,44,45].

Previously, granular BG showed antibacterial properties, but putty made with the BG granules did not show antibacterial properties [46]. This leaves room for developing ABVF-BG putty for harnessing antibacterial as well as bone-healing property. Although, BG may be a good option as a bone void filler due to its bone-healing properties, unlike other BVF materials, conventional casting and melt-derived BG, as in our case, can be challenging to use as antibiotic carriers due to their higher density and lower porosity [47]. However, although, the sol–gel method-derived BG can be used to load antibiotics in their mesoporous structure, they show unpredictable antibiotic release, extremely low overall release (20–25%) of loaded drug, and poor mechanical property [47]. Thus, a BG composite, such as the ABVF-BG putty that we have developed, where BG particles are integrated within a biodegradable polymer matrix that controls drug release, is a viable option for BG-based antibiotic delivery systems for osteomyelitis treatment.

In addition to providing a significant advantage for bone bonding and regeneration, several types of particulate BG such as S53P4 and 45S5 have been shown to possess antibacterial properties in vitro due to increasing pH, osmotic pressure, and calcium ion concentration [48,49]. However, in another apparent disconnect between in vitro performance and in vivo efficacy, particulate BG fillers failed to exert any significant effect on infection prevention in a rabbit open tibial fracture model [50]. Mechanistically, the authors argued that the body’s buffering capacity may have prevented an increase in pH sufficient to lead to bacterial killing. Nevertheless, the efficacy of BG in comparison to calcium sulfate antibiotic beads was assessed in a human trial of osteomyelitis. In that particular trial, the BG showed similar activity as calcium sulfate antibiotic beads; however, it must be noted that both groups of patients received systemic antibiotic for 6–12 weeks as part of the trial, casting doubt on the efficacy of BG as a standalone antimicrobial material [15]. In recent times, S53P4 BG garnered much attention for its antibacterial property and showed so in in vitro studies [51]. Although clinical study did not use local antibiotic when S53P4 BG was used as a bone graft substitute to treat osteomyelitis, patients received systemic antibiotic for 4 to 6 weeks postoperatively [52]. This confounds if S53P4 BG can be used by itself without risking further infection. This is why in our approach, we combined 45S5 BG, for its bone-healing property, with vancomycin for its antibiotic property, so that the combination can be used locally in bone, eliminating systemic side effects of long-term antibiotic therapy.

Several bone graft substitutes, such as calcium sulfate, can be used as antibiotic carriers but create a local acidic environment due to degradation, which subsequently hastens resorption [8]. Other materials such as hydroxyapatite (HA) and tricalcium phosphate, either alone or as a composite, did not show effective antibiotic (vancomycin) release past 21 days [8]. Moreover, HA was shown to have limited resorption in vivo [53]. That is why the current study utilized the BG–polymer composite to deliver vancomycin to provide sustained and effective antibacterial activity, while the BG part was utilized to provide bone-healing property.

Vancomycin still remains the standard recommended antibiotic for treating osteomyelitis caused by *S. aureus* [4]. Moreover, vancomycin is effective against methicillin-resistant *Staphylococcus aureus* (MRSA), and the bacteria is slow to evolve resistance against it [54]. Although the BG in our putty was not shown to have innate antibiotic activity (data not shown), when incorporated into the reported ABVF-BG putty formulation, the drug released from the putty showed antibacterial efficacy against *S. aureus* for up to 6 weeks (Figure 8), which was our targeted timeframe to treat osteomyelitis based on clinical data [6]. Residual antibacterial activity shown after 6 weeks release may be due to some vancomycin still trapped in the remaining polymer matrix. Although not a significant concern for this study, the complete release of drug from the ABVF-BG putty should occur within the desired therapeutic window to avoid inadvertently promoting the development of antibiotic resistant species, which is a consequence that has not been observed with any of our ABVF-BG putty formulations explored to date, as evidenced by the lack of bacterial presence in treatment group animals in in vivo study. Regardless, in the in vivo study, no residual bacterial content could be cultured from the tibia of the treatment group, indicating a significant reduction in bacterial content when compared to the control group, *p* < 0.002. High bacterial content (4.63 × 10^6^ ± 7.9 × 10^5^ CFU/ gram bone) was seen in the tibia harvested from the infection control group implanted with BG putty with no antibiotic in it (Figure 12). In addition, these control rats had a puss-filled capsule at the surgical site, which was separated from the bone and cultured, yielding incredibly high bacterial load, innumerable even after 10^8^ fold dilution.

While the drug release milieu from the ABVF-BG putty effectively killed *S. aureus* both in vitro and in vivo, cytotoxicity is an important criterion to assess. In vitro cell viability assays showed no significant difference in osteoblast cell viability when exposed to day 1 and week 3 drug-released media, although there was a significant dip in viability in the cells treated with week 1 drug-released media (Figure 9). Nevertheless, cell viability remained about 85%, which is within the acceptable limit for medical devices and materials (viability above 70%) according to the International Organization for Standardization guidelines [55]. Interestingly, at day 1 when the drug concentration is anticipated to be the highest (Figure 8) and week 3, which corresponds to the predicted onset of degradation of the putty’s polymer matrix, cell viability was not significantly reduced. Therefore, it is unclear why there was a decline in viability at week 1. The extended systemic use of vancomycin may cause nephrotoxicity [56], which was not seen in our study due to the local sustained delivery of vancomycin. To assess the nephrotoxicity, serum creatinine level was measured, which was found to be within the normal range (Supplementary Figure S1).

In spite of the conundrum surrounding our cytotoxicity assay, the in vitro studies indicated the overall safety and efficacy of our ABVF-BG putty; hence, we proceeded to assess our ABVF-BG putty in vivo, with promising results. X-ray imaging showed healed or healing bone in the treatment group (Figure 10b). In contrast, the infection control group showed the telltale signs of osteomyelitis: deformed bone, osteolysis, periosteal thickening, and formation of sequestrum (Figure 10a). The μ-CT of the bones confirmed the findings. The treatment group bone showed no signs of osteomyelitis, as the bone appeared to be healed with new bone formation and remodeling taking place (Figure 11b). On the contrary, severe osteomyelitis was seen in the infection control group with decreased and deformed bone formation, narrowing marrow space, and the formation of sinus tracts. Infection seemed to be spread to the posterior of the bone, and the presence of a puss-filled capsule was also seen (Figure 11a).

Despite the limited power of this study (only three rats were assessed in each cohort), all three control group rats remained infected at the end of study period with severe osteomyelitis demonstrated, while all the treatment group rats were cured and remained infection free. Although the rate of bone regeneration was not fully assessed during this study, preliminary data (Supplementary Figure S2) and published literature reports suggest that bone regeneration in the presence of BG is enhanced; hence, this remains an open question that should be quantitatively assessed. Nevertheless, bone regeneration and infection abatement were achieved at a satisfactory level in the entire treatment group.

## 4. Materials and Methods

### 4.1. Materials

Poly(D,L-lactide-co-glycolide) 90:10 (PLGA) (Polysciences, Inc., Warington, PA, USA); methylated polyethylene glycol (5kD) (mPEG) (Fluka, St. Louis, MO, USA), and polycaprolactone (10 kD) (PCL-Sigma-Aldrich, St. Louis, MO, USA) were used as received. 45S5 BG powder (particles size d_50_ = 10μm) was obtained from Bonding Chemical (Katy, TX, USA), N-methyl-2-pyrrolidone (NMP) from Fisher Sci (Pittsburg, PA, USA), alamarBlue from Bio Rad (Hercules, CA, USA) were all used as received from the manufacturer. Vancomycin hydrochloride (V-HCl) was obtained from Sagent Pharmaceuticals (Schaumburg, IL, USA). Vancomycin free base (V-fb) was made from V-HCl, as previously described [57].

### 4.2. Fabrication of Bioglass Scaffold

Bioglass (BG) substrate scaffold was fabricated using a modification of a previously described method [16,58]. Briefly, amorphous 45S5 BG particles of approximately 10 μm were suspended (40 wt %) in a polyvinyl alcohol (PVA)–water solution (0.01 mol/L of PVA in water) by vigorously stirring to create a slurry. Subsequently, a polyester-based polyurethane (PU) foam with porosity of 90 ppi and dimensions of 1 cm × 1 cm × 0.5 cm was used as a sacrificial polymer template to fabricate a BG scaffold. The foam was dipped into the BG slurry, letting the particles infiltrate and settle in the porous structure of the PU foam. After soaking for 15 min, excess PVA slurry was squeezed out, and the foam was dried overnight. Then, the dried foams were heated and sintered in a kiln (Paragon Caldera XL Kiln, Clay-King.com, Spartanburg, SC, USA). To sinter the BG scaffolds, the kiln was heated at a rate of 2 °C/min to 400 °C and held at 400 °C for 1 h to burn out the polyurethane foam scaffold. Afterward, the kiln was heated to 1000 °C at 2 °C/min and held at 1000 °C for 1 h. At the conclusion of this incubation period, the kiln was cooled to room temperature at a rate of 5 °C /min (Figure 1). The resulting porous glass structure was collected for further use.

### 4.3. Fabrication of ABVF-BG

The fabricated porous BG scaffold was crushed and sieved to get a particle size distribution of 175 to 425 μm. The BG particles, 350 mg, were soaked in 1 mL of a vancomycin HCl solution (100 mg/mL in phosphate-buffered saline) and dried in a controlled environment (37 °C in a water-jacketed, CO_2_ (5%) incubator, followed by at 50 °C at ambient condition on hot plate). The polymers (PEG, 21.2 mg, and PCL, 42.5 mg) were melted at 65 °C on a steel slide. Antibiotic soaked and dried BG particles were added to the molten polymers and mixed to create a homogenous amalgamation. At this point, vancomycin free base (V-fb, 55.5 mg) was added to the mixture. Subsequently, PLGA, 85.5 mg, dissolved in 200 μL of N-Methyl-2-pyrrolidone (NMP) was mixed into the polymer/drug/BG composite. Later, 20 μL phosphate-buffered saline (PBS) was added dropwise to produce a material with putty-like consistency. This master mix was compressed into a 3D-printed mold to yield a cylindrical shape of 4 mm diameter × 3.5 mm height for further study. The master mix produced 7 cylindrical putties, each weighing 93.5 mg on average.

### 4.4. Characterization of BG Scaffold and ABVF-BG

#### 4.4.1. X-ray Diffraction (XRD)

The sintered scaffolds were manually morselized with a mortar and pestle and used for XRD. As a control, the amorphous BG powder was also used for XRD. A Bruker D8 advanced XRD (Bruker, Billerica, MA, USA) was used, and Cu kα radiation (at 40 kV and 40 mA) was employed. Data were collected over the range of 2*θ* = 0–90° using a step size of 15° and a counting time of 300 s per step.

#### 4.4.2. Scanning Electron Microscopy (SEM) and Energy-Dispersive X-ray Spectrometer (EDS)

For SEM, the samples were attached to cylindrical aluminum mounts with colloidal silver paint (Structure Probe Inc., West Chester, PA, USA) followed by gold sputter coating (Cressington Inc., Redding, CA, USA). Images were taken with a JEOL JSM-6490LV scanning electron microscope. The microscope was equipped with a Thermo Scientific UltraDry EDS detector for recording the EDS spectra.

#### 4.4.3. Assessment of In Vitro Activity in Simulated Body Fluid (SBF)

This study was carried out by following standard methodology as described previously [59]. Briefly, crushed BG particles were washed in deionized (DI) water and allowed to air dry. Then, in a 6-well plate, 100 mg of BG particles were incubated in 4 mL of SBF. Incubation in SBF was for 4 days at 37 °C with exchange of SBF every day. After 4 days, BG particles was rinsed gently in DI water and allowed to dry. Dried and crushed particles were examined using XRD as described in Section 4.4.1 to see any presence of hydroxyapatite (HA) formation.

#### 4.4.4. Micro-Computed Tomography (μ-CT)

For μ-CT, the ABVF-BG was hot glued to a glass rod and placed into a GE Phoenix v|tome| xs X-ray computed tomography system with a 180 kV high power nanofocus X-ray tube xs|180 nf, high contrast GE DXR250RT flat panel detector, and molybdenum target (GE Sensing & Inspection Technologies GmbH, Wunstorf, Germany). One thousand projections were acquired at a voltage of 80 kV and a current of 300 µA. The voxel size was 6.4 µm. Acquired images were reconstructed into a volume dataset using GE datos|x 3D computer tomography software Version 2.2 (GE Sensing & Inspection Technologies GmbH, Wunstorf, Germany). Then, the reconstructed volume was viewed and manipulated using VGStudio Max (Volume Graphics Inc., Charlotte, NC, USA).

### 4.5. In Vitro Antimicrobial Activity Assay

ABVF-BG putty was submerged in PBS (phosphate-buffered saline) release media for drug release study. Cylindrical ABVF-BG (4 mm × 3.5 mm) was put into 2 mL of PBS (1×) in a centrifuge tube and was incubated at 37 °C. At different time points (day 1, day 2, day 3, day 7, and every week after that through 6 weeks), all of the release media were collected and replaced with fresh PBS. After 6 weeks of drug release, the ABVF-BG putty was dissolved in dichloromethane, and residual vancomycin was extracted in deionized (DI) water. In vitro antibacterial activity of the released and residual drug was assessed against *Staphylococcus aureus* (ATCC 49230; the strain is a clinical isolate from a chronic osteomyelitis patient) using a Kirby-Bauer ZOI (zone of inhibition) assay [60]. Briefly, 100 μL of the collected drug release media was dried on a 6.5 mm diameter filter paper disk by incubating at 37 °C. An overnight *S. aureus* culture was prepared and diluted in PBS to get a 10^7^ CFU/mL bacterial concentration. The bacterial solution was streaked on LB agar (Luria–Bertani agar) plates. Subsequently, drug-soaked filter paper disks were placed on the plates (within 15 min of bacteria streaking) and incubated for 20 h at 37 °C. The ZOI around the disk was measured using a digital caliper.

### 4.6. In Vitro Cell Viability Assay

Cytotoxicity of ABVF-BG putty was assessed via a standard alamarBlue assay, following the manufacturer’s protocol. Briefly, 10,000 MG-63 osteoblast cells (ATCC, Manassas, VA, USA) were seeded in each well of a 96-well plate. Cells were grown in Dulbecco’s Modified Eagle Media (DMEM) containing 10% fetal bovine serum (FBS) and 1% penicillin–streptomycin–fungizone (Lonza, Walkersville, MD, USA) and were incubated at 37 °C with 5% CO_2_. After reaching 60% confluency, cells were washed with PBS. Then, 100 μL of release media collected at different time points from ABVF-BG drug release study were added to each well along with 100 μL of cell culture media only (without antibiotics and fungicides). Control wells received 100 μL 1× PBS and 100 μL of cell culture media only. After 48 h of incubation, cells were washed with 1× PBS three times. Fresh media was added to the wells followed by the addition of alamarBlue to a 10% final concentration. Subsequently, wells were incubated at 37 °C for 4 h. After the incubation, the absorbance of the wells was read at 570 nm and 600 nm (Spectramax m5, Molecular Devices, Downingtown, PA, USA). Cell viability was calculated using the following equation:

%cell viability = (((O2 × A1) − (O1 × A2)) ÷ ((O2 × P1) − (O1 × P2))) × 100(1)

where O1 = molar extinction coefficient (E) of oxidized alamarBlue (Blue) at 570 nm, O2 = E of oxidized alamarBlue at 600 nm, A1 = absorbance of test wells at 570 nm, A2 = absorbance of test wells at 600 nm, P1 = absorbance of positive growth control well (cells plus alamarBlue but no test agent) at 570 nm, P2 = absorbance of positive growth control well (cells plus alamarBlue but no test agent at 600 nm).

### 4.7. In Vivo Assessment

#### 4.7.1. Rat Osteomyelitis Model

A pilot animal study was carried out using Sprague–Dawley rats, both male and female (>280 g), with three animals in each of the treatment groups and the control group. The study was approved by the North Dakota State University Institutional Animal Care and Use Committee (IACUC). The approved protocol number was A19019. Briefly, rats were anesthetized by isoflurane inhalation. The right hind leg was shaved and sterilized using alcohol and iodine pads, repeatedly. A small incision of ≈12 mm was made below the knee over the tibial metaphysis. A 4.2 mm hole was drilled using an orthopedic drill (Stryker, Kalamazoo, MI, USA) until it penetrated the bone and revealed the marrow space of the tibial metaphysis. Overnight *S. aureus* culture diluted in sterile saline to achieve a concentration of 10^8^ CFU/10 μL was injected (10 μL) through the drill hole defect into the marrow space using a 25 μL Hamilton syringe followed by implantation of the ABVF-BG putty to fill up the drilled hole. The incision was closed using a series of mattress sutures followed by application of surgical glue (Vetbond Tissue Adhesive, 3M, St. Paul, MN, USA). Following the surgery, buprenorphine hydrochloride (Hospira, Inc., Lake Forest, IL, USA) was injected (0.01 mg/kg) subcutaneously as an analgesic. The control group underwent the same surgical procedure but received putty without antibiotic (BVF putty). Rats were assigned randomly to the control and treatment group. Rats were monitored daily for signs of discomfort and infection. After 8 weeks, the rats were sacrificed, and the tibia was harvested for further study.

#### 4.7.2. X-ray and μ-CT of Rat Bones

Following euthanasia, radiographic analysis of bones was done after disarticulating the limb and removing the soft tissue to isolate the bone. X-ray imaging was done using an IDEXX CR Digital Radiography System (Westbrook, ME, USA) following standard protocols. Briefly, lateral and cranial–caudal radiographic images of each limb were obtained at mAs: 4 and kVp: 40. The μ-CT of the bone was done following a similar procedure as described in Section 4.4.4 with slight adjustment of current to 400–500 µA.

#### 4.7.3. Bacterial Colony Count

Harvested bone was flash frozen in liquid nitrogen, which was followed by pulverization using a custom-made bone crusher. The pulverized bone was weighed and suspended in 500 μL of PBS. Serial dilutions were made [57], and 10 μL of suspension was plated on blood agar plates (Fisher Sci, Pittsburg, PA, USA). Subsequently, plates were incubated for 48 h at 37 °C, and bacterial colonies were counted. To get the total number of bacteria in the total sample, the number of colonies were multiplied by the dilution factor and normalized per gram of bone.

#### 4.7.4. Histology

After euthanasia, the bone was harvested and fixed in 10% neutral buffered formalin for 72 h. Subsequently, bone was decalcified by immersing it in an EDTA solution (10% solution at pH 7.4) for 2 weeks. The EDTA solution was exchanged every other day. Once the bone was decalcified, it was embedded in paraffin wax and sectioned (5 μm). Sections were mounted on glass slides and stained with hematoxylin and eosin stains (H&E stainScy Tek Lab., Logan, UT, USA) according to standard protocols. Briefly, the sections were deparaffinized in Clear Rite 3 (Thermo Fisher Scientific, Kalamazoo, MI, USA). Subsequently, tissue was rehydrated with a decreasing gradient of ethanol. After H&E staining, the tissue section was covered with a glass coverslip using synthetic resin mounting media (Cytoseal XYL, Thermofisher, Kalamazoo, MI, USA). Stained slides were imaged at 40x using MoticEasyScan Digital Slide Scanning microscope (Motic Digital Pathology, San Francisco, CA, USA).

### 4.8. Statistical Analysis

The statistical package in Microsoft Excel 2016 (Microsoft Corp., Redmond, WA, USA) was used for all calculations and statistical analyses. A Student’s T-test using α = 0.05 was done to determine statistical significance. The XRD data were plotted and analyzed using OriginPro 8.5 (OriginLab, Northampton, MA, USA).

## 5. Conclusions

The successful infection treatment and satisfactory bone healing in rats shows the promise of a BG-based ABVF-BG putty to treat osteomyelitis. ABVF-BG provided effective release of vancomycin showing in vitro and in vivo efficacy against *S. aureus*. ABVF-BG was biodegradable and provided support for new bone growth. No adverse effects were seen on either overall rat health or on in vivo bone healing. The BG-based ABVF-BG putty developed in this study can be a promising option for difficult to treat osteomyelitis.

## Supplementary Materials

The following are available online at https://www.mdpi.com/article/10.3390/ijms22147736/s1.
