# Supplementary material for: A Bioglass-Based Antibiotic (Vancomycin) Releasing Bone Void Filling Putty to Treat Osteomyelitis and Aid Bone Healing
Source: Int J Mol Sci. 2021 Jul 20;22(14):7736. doi: 10.3390/ijms22147736 (PMC8304857; doi:10.3390/ijms22147736)
Supplement: Supplementary file 1 [file ijms-22-07736-s001.zip › ijms-1239258-supplementary.pdf]

International Journal of *Molecular Sciences*

MDPI

Supplementary Materials: Bioglass based Antibiotic Releasing Bone-void Filling Putty to Treat Osteomyelitis and to Aid Bone Healing

Raquib Hasan [1,*], Kambri Schaner [2], Pranothi Mulinti [1] and Amanda Brooks [1,3,*]

[1] Department of Pharmaceutical Sciences, North Dakota State University, Fargo, ND 58102, USA; raquib.hasan@ndsu.edu (R.H.), pranothimulinti@gmail.com (P.M.)
[2] Department of Biological Sciences, North Dakota State University, Fargo, ND 58102, USA ; kambri.schaner@ndsu.edu
[3] Office of Research and Scholarly Activity, Rocky Vista University, Ivins, UT 84734, USA; abrooks@rvu.edu
* Correspondence: abrooks@rvu.edu (A.B.); raquib.hasan@ndsu.edu (R.H.)

S1 Serum Creatinine

Blood was collected from the treatment, control group rats and no-surgery control rats. Collected blood samples were centrifuged at 2000 g (Allegra X-14R, Beckman Coulter, Brea, CA, USA) to collect the serum. The serum was used to measure the creatinine level using an enzymatic rat creatinine assay kit (Crystal Chem, IL, USA) following the manufacturer's protocol.

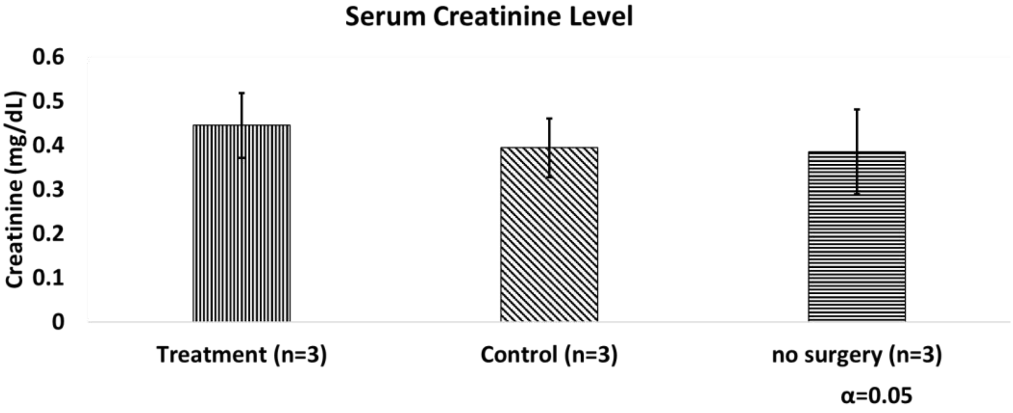

Figure S1. Serum creatinine levels show no significant difference ($\alpha = 0.05$) between the treatment (n = 3), control (n = 3), and non-surgical (no infection) control groups (n = 3). The serum creatinine levels were within normal limits.

S2 Bone Regeneration after Implanting ABVF-BG in Rat Bones

In a preliminary assessment, ABVF-BG putty, described on the current article, or, ABVF putty containing HA and calcium carbonate based particles as bone graft substitute instead of bioglass particles, described in a previous article [1] was implanted in rat tibia after drilling hole. The rats in ABVF-BG no-infection control group rats were euthanized at week 6 (n=1) and week 10 (n=2) and ABVF no-infection control group rats were euthanized at week 10 (n=3) and μ-CT scan was done on the harvested bone to assess the volume of newly generated bone. One treatment group rat from the current study also assessed for new bone formation after euthanasia at 8 weeks. The ABVF-BG no infection control group rat showed more new bone formation at 6 weeks compared to ABVF no infection control group at 10 weeks. This indicates faster bone formation in ABVF-BG group rat bones. Between the two types of putty implants, only bone graft substitutes varied, and the putty containing bioglass showed faster and higher bone formation indicating that bioglass helps in faster bone formation. In the ABVF-BG no infection control group, at week 10, not much new bone formed compared to week 6. That may indicate

that the new bone formation was achieved earlier and then it might have plateaued. The treatment group rats showed good bone formation at week 8. It would be interesting to see a comparison of new bone formation due to the two different putty implants and how they impact the bone regeneration a in a full powered animal study. It should be noted here that these observations are preliminary and was not a part of full powered animal study.

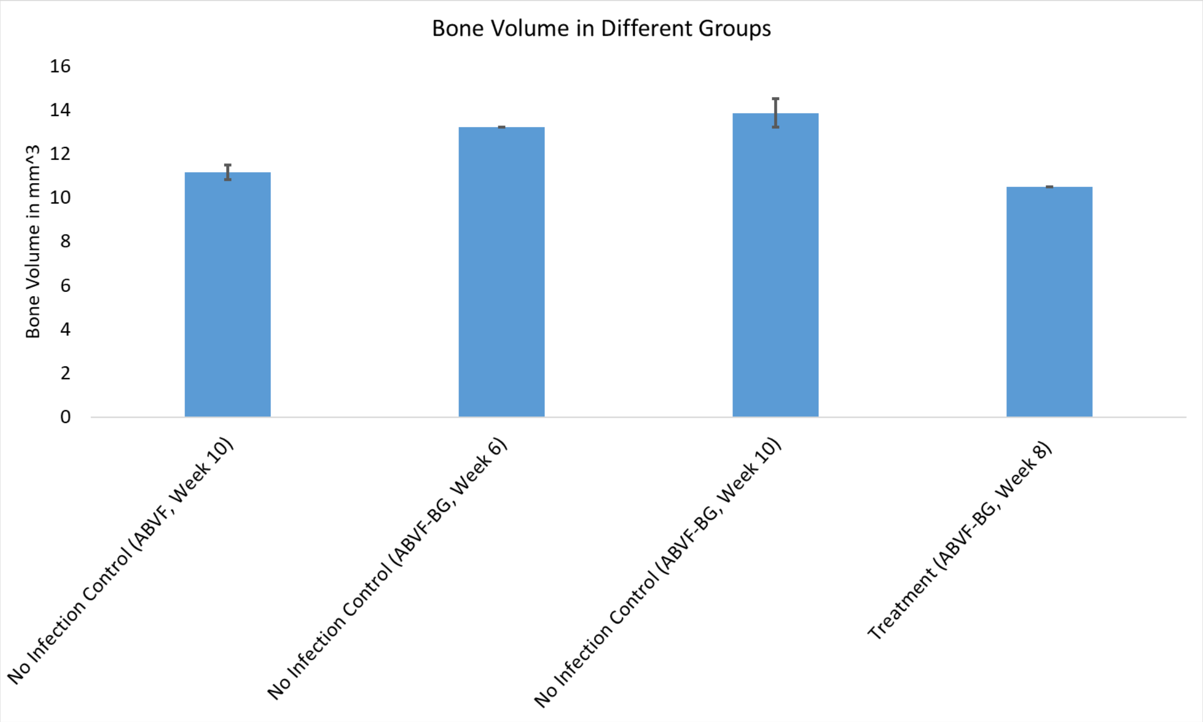

Figure S2. Rats receiving ABVF-BG putty showed faster and higher new bone growth compared to the rats receiving ABVF that contained HA and calcium carbonate based particles instead of bioglass particles. The ABVF-BG treatment rat showed similar level of new bone regeneration as the no infection ABVF control rats in less time.

Reference:

1. Hasan, R.; Wohlers, A.; Shreffler, J.; Mulinti, P.; Ostlie, H.; Schaper, C.; Brooks, B.; Brooks, A. An Antibiotic-Releasing Bone Void Filling (ABVF) Putty for the Treatment of Osteomyelitis. *Materials* **2020**, *13*, 5080, doi:10.3390/ma13225080.
